# Upper- and lower-limb amputees show reduced levels of eeriness for images of prosthetic hands

**DOI:** 10.3758/s13423-019-01612-x

**Published:** 2019-06-10

**Authors:** Gavin Buckingham, Johnny Parr, Greg Wood, Sarah Day, Alix Chadwell, John Head, Adam Galpin, Laurence Kenney, Peter Kyberd, Emma Gowen, Ellen Poliakoff

**Affiliations:** 1 Department of Sport and Health Sciences, University of Exeter, Richards Building, Exeter, EX1 2LU UK; 2 Department of Health Sciences, Liverpool Hope University, Liverpool, UK; 3 Research Centre for Musculoskeletal Science and Sports Medicine, Manchester Metropolitan University, Manchester, UK; 4 National Centre for Prosthetics and Orthotics, Department of Biomedical Engineering, University of Strathclyde, Glasgow, UK; 5 Centre for Health Sciences Research, University of Salford, Salford, UK; 6 School of Energy and Electronic Engineering, University of Portsmouth, Portsmouth, UK; 7 Division of Neuroscience and Experimental Psychology, University of Manchester, Manchester, UK

**Keywords:** Uncanny valley, Uncanny phenomenon, Prosthetic use, Perception, Embodiment

## Abstract

The “uncanny phenomenon” describes the feeling of unease associated with seeing an image that is close to appearing human. Prosthetic hands in particular are well known to induce this effect. Little is known, however, about this phenomenon from the viewpoint of prosthesis users. We studied perceptions of eeriness and human-likeness for images of different types of mechanical, cosmetic, and anatomic hands in upper-limb prosthesis users (n=9), lower-limb prosthesis users (n=10), prosthetists (n=16), control participants with no prosthetic training (n=20), and control participants who were trained to use a myoelectric prosthetic hand simulator (n=23). Both the upper- and lower-limb prosthesis user groups showed a reduced uncanny phenomenon (i.e., significantly lower levels of eeriness) for cosmetic prosthetic hands compared to the other groups, with no concomitant reduction in how these stimuli were rated in terms of human-likeness. However, a similar effect was found neither for prosthetists with prolonged visual experience of prosthetic hands nor for the group with short-term training with the simulator. These findings in the prosthesis users therefore seem likely to be related to limb absence or prolonged experience with prostheses.

## Introduction

The “uncanny valley” describes the experience of unease or repulsion in the presence of an object that falls just short of being human (Mori, 1970). As implied by the term, the effect is not a linear one – individuals typically feel more affinity for artificial objects as they become more human-like, before reporting a sharp drop-off in their levels of affinity (accompanied by disgust or unease – MacDorman & Ishiguro, 2006). However, uncertain findings related to the shape of the valley’s “distribution,” combined with the obvious difficulties in defining human-likeness, has led some researchers to adopt the term “uncanny phenomenon” to describe the feeling of unease associated with broadly human-like stimuli, without making any assumptions as to the dimensionality of the underpinning distribution (Wang, Lilienfeld, & Rochat, 2015). The mechanism underpinning this phenomenon is widely debated, but recent studies suggest that it might represent the conflict between a biological appearance and other features, such as temperature, hardness, and exhibiting non-biological kinematics (Kätsyri, Förger, Mäkäräinen, & Takala, 2015; Saygin, Chaminade, Ishiguro, Driver, & Frith, 2012). The “uncanny phenomenon” is most frequently experienced in the context of computer-generated animations of human faces, or interactive robots, and thus much of the research in the topic is focused on computer-science domains (Destephe et al., 2015; MacDorman, Green, Ho, & Koch, 2009). An important healthcare domain where the uncanny phenomenon has increasing relevance is in the development of prosthetic limbs (Cabibihan et al., 2006). The relevance of the uncanny phenomenon to prosthetic hands was strong enough for Mori, in his original article (1970), to even suggest that prosthetic designers eschew life-like materials altogether when developing new limbs.

Despite these early suggestions, it is only recently that there have been empirical reports showing that participants rate life-like prosthetic hands to be eerier than either mechanical hands or anatomic human hands (Poliakoff, Beach, Best, Howard, & Gowen, 2013; Poliakoff, O’Kane, Carefoot, Kyberd, & Gowen, 2018). Both of these studies, however, have been undertaken in populations without any significant experience of upper-limb prostheses (i.e., university students), and no empirical work has examined the degree to which prosthetic limb users experience this phenomenon. Indeed, little is known at all about how experience with the target stimuli affects the uncanny phenomenon. A recent study in the context of human-robot interaction suggests that repeated interactions with a lifelike robot appeared to reduce the feelings of unease towards it (Burleigh & Schoenherr, 2014; Złotowski et al., 2015). How these findings would generalize to the use of an upper-limb prosthesis is, however, unclear. Prosthesis users, perhaps unsurprisingly, generally express a preference for life-like devices, particularly in the context of the upper limbs (Biddiss, Beaton, & Chau, 2007). Prosthetic limb users, of course, represent a group who have a particular type of experience, having actively used a prosthetic limb, possibly embodying it (Murray, 2004; Niedernhuber, Barone, & Lenggenhager, 2018). Indeed, there have been reports that the use of a prosthetic limb fundamentally changes one’s haptic experience of object weight (Buckingham et al., 2018), categorization ability (van den Heiligenberg, Yeung, Brugger, Culham, & Makin, 2017), and even visual perception (Nico, Daprati, Rigal, Parsons, & Sirigu, 2004).

Our goals with the current study were twofold. First, we aimed to examine the degree to which upper-limb prosthesis users experience the uncanny phenomenon for prosthetic hands. Second, we hoped to further the understanding of how different types and extents of practical experience with prostheses might affect the uncanny phenomenon in a range of populations, including groups with limb absence and anatomically intact groups. To this end, we used the hand stimuli developed by Poliakoff et al. (2018) to examine ratings of eeriness and human-likeness in upper-limb prosthesis users, lower-limb prosthesis users, prosthetists (individuals who are involved in fitting prosthetic limbs), anatomically intact controls using their anatomic limb, and anatomically intact controls using an upper-limb prosthesis simulator on which they had received extensive training. These different groups were examined due to their differing and dissociable levels of visual experience with, and practical use of, prosthetic hands. We expected the control group to broadly replicate the patterns found in the work of Poliakoff et al. (2018), showing the strongest levels of unease for the unrealistic-looking covered prosthetic hands. We predicted that the upper-limb prosthesis users would be the least affected by the uncanny phenomenon, due to both their visual familiarity with the subjects of the images and the use of their own prosthetic limbs. We predicted the lower-limb prosthesis users would also have a significant degree of general prosthesis experience, without the active use of a prosthetic hand, which might make them similarly less prone to the uncanny phenomenon if visual experience is a key factor in the effect. Similarly, we reasoned that the prosthetists would have a significant amount of experience with prosthetic hands, but without experiencing any meaningful embodiment of the hands. By contrast, the group trained with a prosthesis simulator would have relatively little visual experience compared to the non-control groups, but a relatively large degree of experience actively using a prosthesis compared to all except the upper-limb prosthesis group.

## Method

### Participants

We tested five groups of participants in this study, each with varying levels of experience with upper-limb prostheses. Our first group comprised nine prosthesis users with upper limb absence, recruited through the Universities of Strathclyde and Salford (eight male, mean age = 62.6 years, SD = 11.2). The individuals in this group used a prosthesis as a result of either congenital or acquired limb absence, but all used an upper limb prosthesis regularly. Our second group was made up of ten lower-limb prosthesis users (all male, mean age = 59.8 years, SD = 14.8). Our third group was made up of 16 practicing prosthetists and final-year prosthetics students, recruited from the University of Strathclyde (three male, mean age = 22.1 years, SD = 2.6). Our fourth group was 24 anatomically intact university students (12 male, mean age = 24.5 years, SD = 7.4) recruited from Liverpool Hope University, who had received 6 h training over a 2-week period in a visuomotor task using a BeBionic (Otto Bock HealthCare, Duderstadt, Germany) myoelectric prosthetic hand simulator (for details, see Parr, Vine, Harrison, & Wood, 2018). The final group consisted of 20 further anatomically intact university students (controls) recruited from Manchester Metropolitan University (13 male, mean age = 25.8 years, SD = 8.3), in a similar protocol to that outlined by Poliakoff et al. (2018).

All participants gave written informed consent prior to testing, and all procedures were approved by the local research ethics boards at the University of Strathclyde, Liverpool Hope University, and Manchester Metropolitan University.

### Materials and procedure

Participants rated the eeriness and human-likeness of the 12 photographic images shown in Fig. 1a of Poliakoff et al. (2018). In brief, these images consist of three robotic hands (hereafter referred to as “mechanical”), three unrealistic-looking prosthetic hands, three realistic-looking prosthetic hands, and three anatomic human hands. All hand stimuli were right hands, posed at roughly the same relaxed posture with the wrist down against a neutral black background. Stimuli were presented sequentially on a laptop screen using Microsoft Powerpoint until a verbal response was given. First, participants were asked to verbally rate each of the images on a 9-point Likert scale in terms of “how eerie is this hand,” with 0 being “not at all” and 9 being “extremely.” Each image was rated twice, for a total of 24 ratings, in one of three pseudorandomly-generated orders. Next, participants were asked to rate “how human-like is this hand” on the same scale. As per the eeriness ratings, each hand was rated twice for 24 ratings in total (in the same random order as the eeriness ratings). Eeriness was defined as “mysterious, strange, or unexpected so as to send a chill up the spine” and human-likeness as “having human form or attributes.” All 48 ratings (recorded by the experimenter) were given in a single session lasting approximately 30 min. For the Trained group, the experiment took place immediately following their final training session using the prosthesis simulator.

**Fig. 1:**
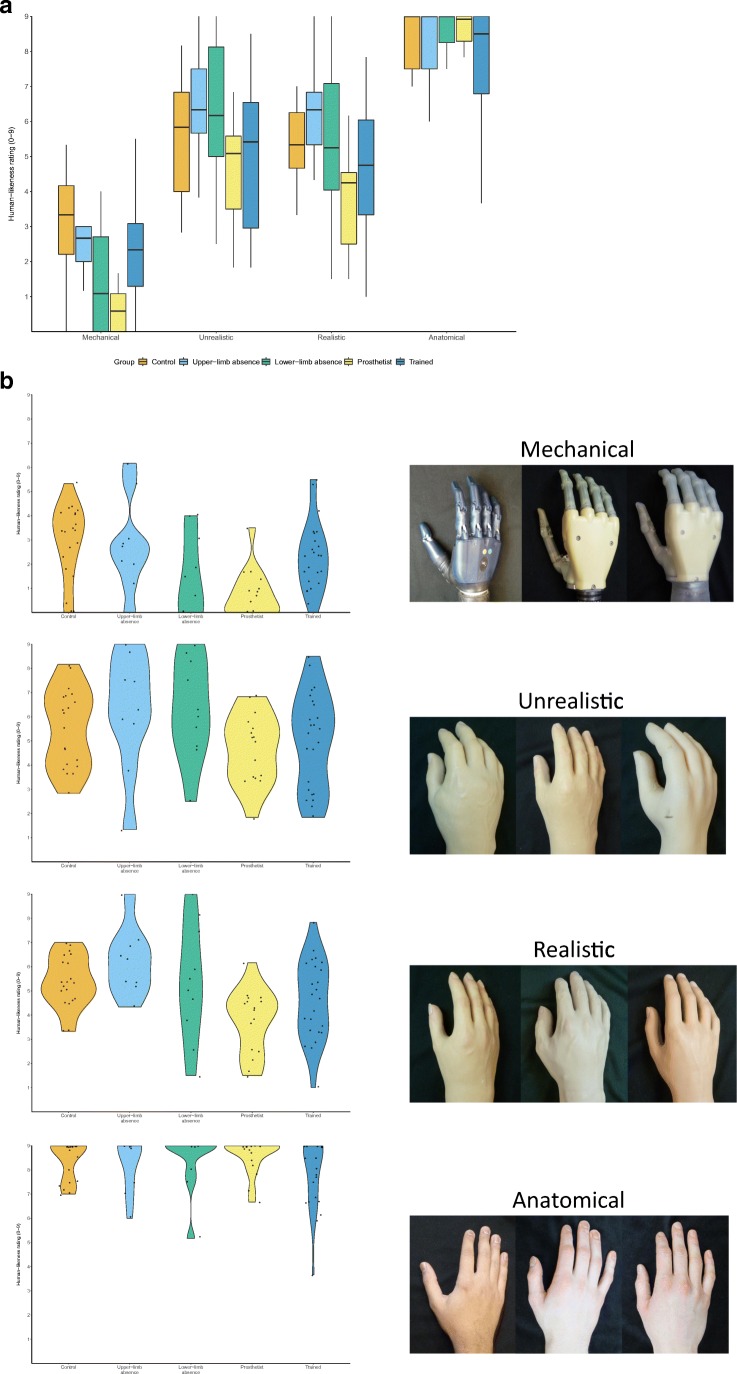
(**a**) Median human-likeness ratings for the different hand types for each group. Higher numbers indicate that participants reported that the hand appeared more human-like. Boxes show quartiles and tails show 95% confidence intervals. (**b**) The human-likeness ratings given by the members of each group in each condition, presented as individual violin plots to better visualize the distributions of the data

The two ratings for each hand were averaged, and the average ratings of the three photographs in each condition were examined in separate 4 x 5 mixed ANOVAs with four repeated levels (hand type: Mechanical, Unrealistic, Realistic, Anatomical) and five between-group levels (group: Upper-limb absence, Lower-limb absence, Prosthetist, Trained, Control) for each measure. The Greenhouse-Geisser correction was employed for violations of sphericity. Significant interactions were followed with separate Kruskal-Wallis tests, followed by Dwass-Steel-Critchlow-Fligner pairwise comparisons due to the non-parametric distribution of the measures. Statistical analysis was performed in JAMOVI 0.9.2.3, and an alpha of .05 was used to indicate statistical significance. Average ratings given by each participant can be found here: https://osf.io/ut3ge/.

## Results

### Human-likeness

In terms of our measure of human-likeness, we observed a significant main effect of Hand type (F(2.3,169.9)=314.9, p<.001, η^2^=0.782) and a significant main effect of Group (F(4,74)=3.6, p=.01, η^2^=0.153). We also observed a significant interaction between our factors (F(9.2, 169.9)=3.41, p<.001, η^2^=0.034), as shown in Fig. 1a.

Examining ratings of human-likeness of the mechanical hands in isolation with the Kruskal-Wallis test we observed a significant effect of Group (χ^2^(4)=21.1, p<.001), with Dwass-Steel-Critchlow-Flinger pairwise comparisons showing that the prosthetist group reported that the mechanical hands appeared less human-like than the control group (p<.001), the trained group (p<.001), or the upper-limb absence group (p=.004). Additionally, the control group rated the mechanical hands as less human-like than the lower-limb absence group (p=.027), but no other significant differences were observed. There was no significant effect of group observed for the unrealistic hands (χ^2^(4)=7.47, p=.11). There was, however, a significant effect observed for the realistic hand stimuli (χ^2^(4)=17.62, p=.001), with pairwise comparisons showing that the prosthetist group rated the realistic hands as less human-like than the control group (p<.001) or the upper-limb absence group (p<.001), with no other significant differences observed. Finally, no differences were observed between the groups in terms of how human-like they rated the anatomic hands (χ^2^(4)=2.37, p=.67).

In summary, the plots (Fig. 1b) and associated analyses suggest that prosthetists tend to report some hand types as being less human-like than most other groups.

### Eeriness

**Fig. 2:**
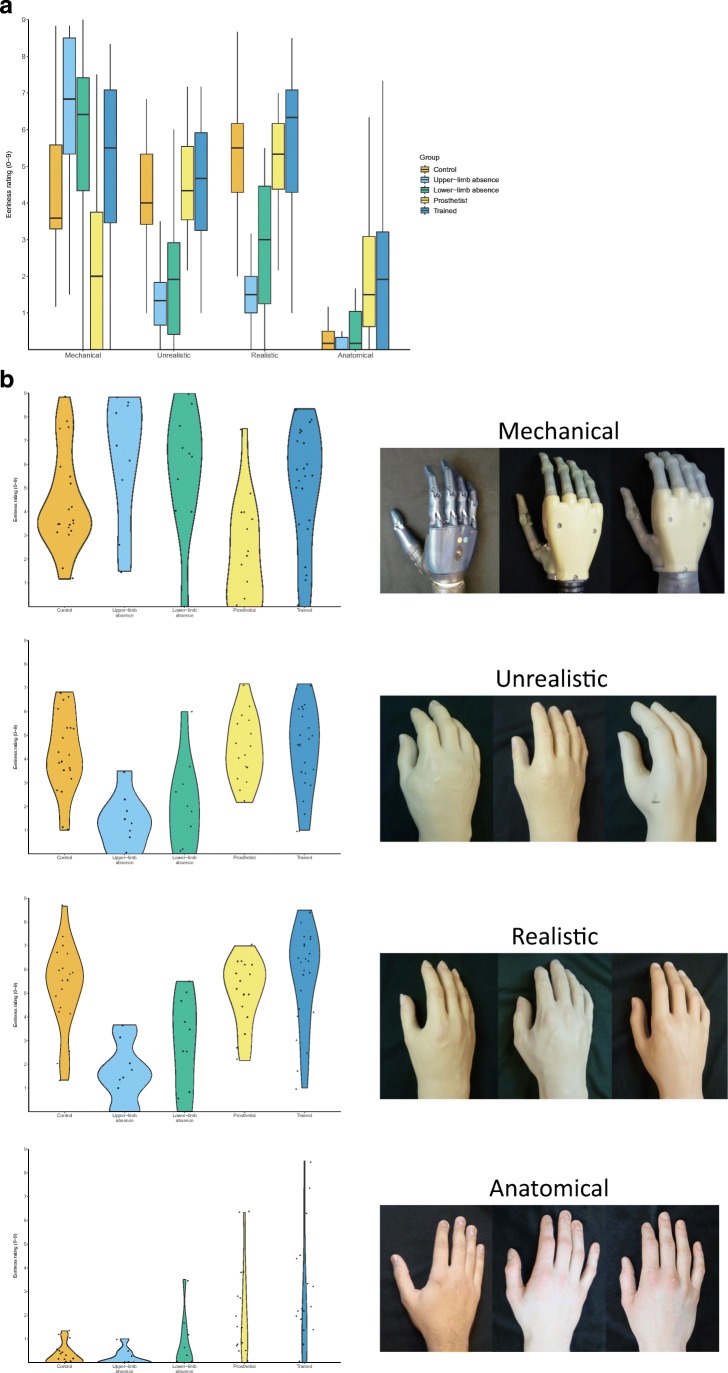
(**a**) Median eeriness ratings for the different hand types for each group. Higher numbers indicate that participants reported the hands to be more eerie. Boxes show quartiles and tails show 95% confidence intervals. (**b**) The eeriness ratings given by the members of each group in each condition, presented as individual violin plots to better visualize the distributions of the data

In terms of our eeriness measure, we observed a significant main effect of Hand type (F(1.91,141.22)=57.97, p<.001, η^2^=0.342) and a significant main effect of Group (F(4,74)=5.42, p<.001, η^2^=0.227). We also observed a significant interaction between our factors (F(7.6, 141.22)=9.34, p<.001, η^2^=0.221), as shown in Fig. 2b.

Examining ratings of the mechanical hands in isolation we observed a significant effect of group (χ^2^(4)=18.3, p=.001), with Dwass-Steel-Critchlow-Flinger pairwise comparisons showing that our prosthetists rated mechanical limbs as less eerie than participants with upper-limb absence (p=.002), those with lower-limb absence (p=.003), the control group (p=.006), or the trained group (p=.002), with no other significant differences observed.

Comparing ratings given by each group to the unrealistic hands also yielded a significant effect (χ^2^(4)=24.8, p<.001). Here, in contrast to the mechanical hand ratings, pairwise comparisons of the unrealistic-looking hands highlighted that both the lower-limb and upper-limb absence groups found this prosthesis type significantly less eerie than the controls (p=.005 and p<.001, respectively), the prosthetists (p=.003 and p<.001, respectively), or the trained group (p=.004 and p<.001, respectively), with no other significant differences observed.

The ratings of the realistic prosthetic hands yielded a similar pattern (χ^2^(4)=28.7, p<.001), with the lower-limb and upper-limb absence groups reporting the realistic hand to be less eerie than controls (p=.003 and p<.001, respectively), prosthetists (p=.031 and p=.002, respectively), or trained individuals (p<.001 in both cases), with no other significant differences observed.

With regards to the anatomic hand, where no differences were predicted, we in fact noted a significant effect (χ^2^(4)=19.4, p<.001). Here, pairwise comparisons showed that the controls rated the anatomic hands as looking less eerie than the trained groups (p=.007) or the prosthetists group (p<.001), who themselves rated these stimuli as more eerie than the upper-limb absence (p=.002) or the lower-limb absence groups (p=.03). No other significant differences were observed between the groups.

In summary, the plots (Fig. 2b) and associated analyses suggest that both upper-limb and lower-limb absence groups rated the realistic and unrealistic prosthetic hands (the type predicted to induce the highest levels of eeriness) as being significantly less eerie than most other groups of participants tested in this study.

## Discussion

Here, we investigated the uncanny phenomenon – the experience of unease when a stimulus falls just short of being human – for prosthetic hands in individuals with distinct levels of experience with prostheses. We compared the ratings of eeriness and human-likeness for images of mechanical, unrealistic, realistic, and anatomic hands for a group of university students, a group of upper-limb prosthesis users, a group of lower-limb prosthesis users, a group of prosthetists, and a group of anatomically intact subjects who had received extensive training on a myoelectric prosthesis simulator.

Prior work with our stimulus set (Poliakoff et al., 2018) led us to predict that participants without any significant experience of prosthetic hands would find the unrealistic cosmetic prostheses more eerie than the mechanical hands, which they would in turn rate as more eerie than the anatomic hands (i.e., an “uncanny valley”). Our control group, however, seemed to experience all the types prosthetic hands as being equally eerie (Fig. 2), despite clear differences in how human-like these hands appeared to this group (Fig. 1) – findings that are analogous to those that have led some researchers (e.g., Wang et al., 2015) to adopt the more neutral “uncanny phenomenon” nomenclature we refer to throughout our paper. The reason for the apparent discrepancy between the current work and those of Poliakoff et al. (2018) is not immediately clear, but may be due to differences in how the task was administered between the studies. To evaluate whether our control group may be performing abnormally compared to the larger sample reported in previous work, we compared the eeriness ratings given by the sample in Poliakoff et al.’s (2018) study and the control group in the current work with an uncorrected independent samples t-test, finding no differences for any of the stimuli sets (all p-values > .23). Furthermore, and unexpectedly, all groups except for the prosthetist group rated the mechanical hands as inducing high levels of eeriness – a finding that may be due to the experience and insights that prosthetists might have gained with the underlying mechanisms of such robotic devices over the course of their training, with such concrete and mechanistic understanding going some way to reducing the expectancy violations that may underpin the uncanny phenomenon (Saygin et al., 2012).

We found clear evidence that the type of experience with a prosthesis affects the uncanny phenomenon with this type of stimulus. This study is the first to show that both upper- and lower-limb prosthetic limb users found the realistic and unrealistic cosmetic prostheses to be significantly less eerie than the other groups, despite no difference in the ratings of human-likeness for these stimuli. With regards to the people with upper-limb absence, it may be that this effect is simply related to visual familiarity – it is one of the most reliable findings in psychology that exposure to a stimulus increases one’s liking toward it (Bornstein, 1989). Given that all of the individuals in the upper-limb absence group were long-term users of an upper-limb prosthesis, who would have had considerably more experience with these types of limbs than any of the other groups, any inherent feeling of eeriness or disgust may have been mitigated by this visual familiarity. This suggestion is consistent with recent work in the context of human-robot interaction, which has suggested that prolonged exposure appears to reduce the sensation of unease induced by a lifelike robot designed to induce the uncanny phenomenon (Złotowski et al., 2015). It is less easy, however, to explain this effect in the lower-limb prosthesis users, who seem unlikely to have had more visual exposure to an upper-limb prosthetic device than members of the prosthetist group. It could be speculated that the general use of a prosthesis, or indeed the absence of a limb, renders an individual less susceptible to the uncanny phenomenon by virtue of having weaker prior expectations of the associations between biological images and non-biological features, and thus less of a response to violations of this relationship (Saygin et al., 2012). This is clearly is a topic that warrants further study, given prosthesis users’ diverse range of preferences for anatomically-realistic prosthetic hands versus the “high-tech” appearance of mechanical prosthetic hands (Biddiss et al., 2007; Kyberd & Hill, 2011).

It is worth evaluating what can be learned from comparisons of the various anatomically intact groups to the control group. Our group of prosthetists was included to allow us to evaluate whether “hands-on” experience with prostheses might modulate the experience of the uncanny phenomenon. Although this group did tend to rate the mechanical prosthetic hands as less eerie than their counterparts, and rated all non-anatomic hands as appearing to be less human, they showed no such effects with images of the cosmetic prostheses. This finding suggests that the effects outlined above are specific to either extremely long-term exposure, the absence of a limb, or the use and embodiment of a prosthetic limb (of any sort). The latter proposition is not, however, strongly supported by the finding that the group who had trained with a prosthetic simulator experienced no reduction in their uncanny phenomenon in any condition compared to controls (notably the mechanical hand condition, which was the closest match to the prostheses on which they trained). Follow-up work with stimuli tailored to the specific prosthesis, in addition to studies examining perception of the uncanny phenomenon in amputees who do not regularly use a prosthesis, will allow us to draw more concrete conclusions. It should also be noted that, unexpectedly, both the prosthetist and the trained group rated the anatomic hands as more eerie than other groups. The conclusion that experience with artificial hands could affect perception of real hands, if replicated, could a fruitful topic of study for future research.

The current work examined the uncanny phenomenon in a diverse range of individuals, which comes with several necessary caveats. For one, the prosthesis users were substantially older than our other groups. We know of no research, however, suggesting that the uncanny phenomenon tends to reduce over the lifespan and, given that this phenomena appears stable from 12 months of age (Lewkowicz & Ghazanfar, 2012), we feel this factor is unlikely to play a major role in our findings. Similarly, our groups were not well matched for gender split, and at least one study to date has noted that males tend to rate androids as less eerie than females rating the same stimuli (MacDorman & Entezari, 2015). Finally, it is worth noting that no effort was made to match up the upper-limb-absent or trained prosthesis users’ prosthesis with the image they were rating – showing a stronger association of this nature would provide particularly compelling evidence for our conclusions that long-term experience with a prosthesis can modulate feelings of unease in this context.

In summary, we have shown that people with upper- and lower-limb absence who use a prosthetic limb have reduced feelings of unease associated with images of life-like cosmetic prosthetic hands, which characterizes the uncanny phenomenon. This reduction of the uncanny phenomenon was not seen in a group of prosthetists or intact individuals who had received training to use a prosthetic simulator, suggesting that this effect might be specific to limb absence or long-term experience with prosthetic limbs.

**Open practices statement**

The data for this experiment are available at https://osf.io/ut3ge/. The experiment was not pre-registered.
